# Supplementary material for: Apparent competition drives community-wide parasitism rates and changes in host abundance across ecosystem boundaries
Source: Nat Commun. 2016 Aug 31;7:12644. doi: 10.1038/ncomms12644 (PMC5013663; doi:10.1038/ncomms12644)
Supplement: Supplementary Information — Supplementary Figures 1-4, Supplementary Tables 1-12 and Supplementary References [file ncomms12644-s1.pdf]

Supplementary Figures

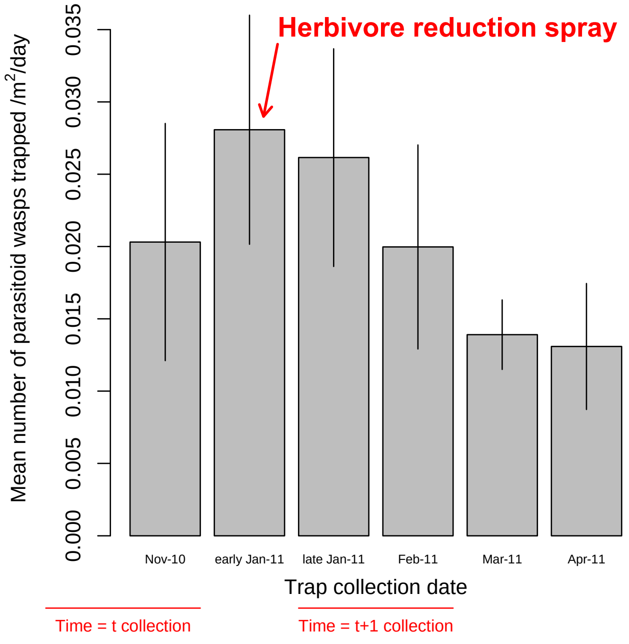

Supplementary Figure 1. Timing of experiment and sampling relative to parasitoid activity.. We attempted to time the herbivore reduction spray at the validation sites roughly so that it would be between larval parasitoid generations, such that most parasitoids present at our sites would be in adult form, and thus not killed along with their hosts during the herbivore reduction spray treatment. That is, the adult parasitoids from the first generation would have emerged, and would be mating and ovipositing at the time of the herbivore reduction treatment. We present flight intercept trap data collected during this study but analyzed elsewhere[1] for adult parasitoid wasps (Ichneumonidae and Braconidae) of the same species as were reared from caterpillars in this study. Flight intercept traps at control sites were used to monitor parasitoid wasp movement across the edge between plantation and native forest at each site[1]. Our herbivore reduction spray occurred at the point in the summer when adult parasitoid wasp activity was highest (i.e. most had emerged as adults and were moving around mating and ovipositing), and thus should have

killed relatively few parasitoid larvae of the first or second generations. Our caterpillar collections to obtain quantitative food web data at the validation sites at time *t* and *t*+1 occurred before and after the herbivore reduction spray respectively, as marked below the x-axis in red, with lines representing the time periods over which each collection occurred.

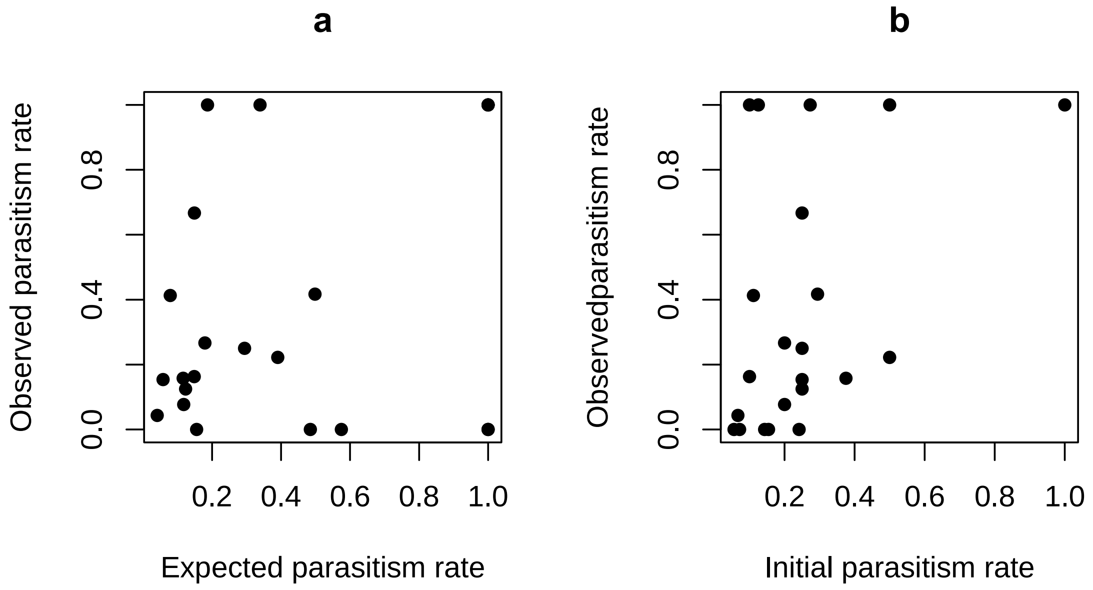

Supplementary Figure 2. Raw data corresponding to Fig. 5a,b. Here we present the raw data for which statistical models showed that (a) expected parasitism rate significantly predicts observed parasitism rate, but (b) initial parasitism rate does not. Fig. 5a,b presents the same data, but with a random effect removing the variation among sites. Each point represents a species within a site that was collected and successfully reared at both time steps, and was parasitized in the first time step (see Eq. 3, Fig. 1i).

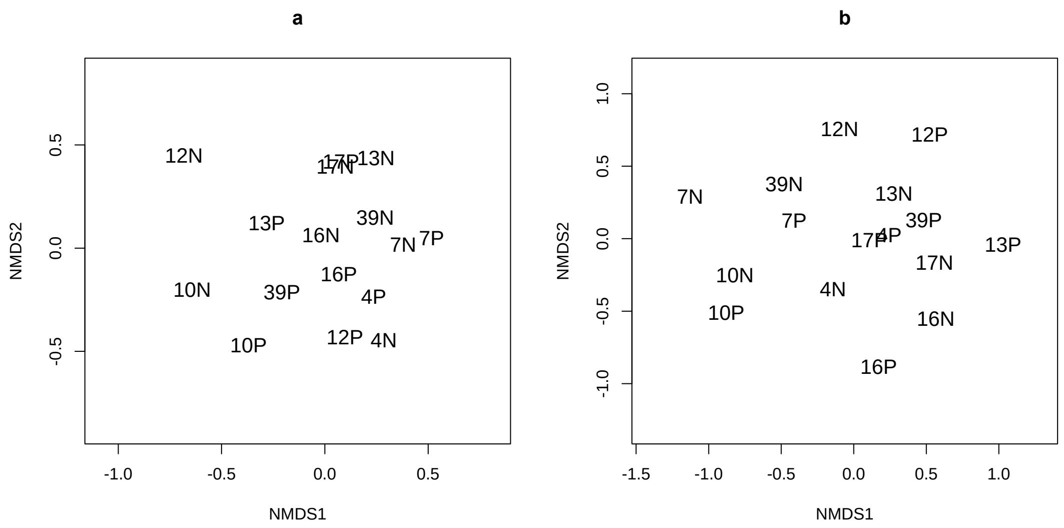

Supplementary Figure 3. Host and parasitoid species composition in different forest types. (a) Host (Lepidoptera) species composition, and (b) parasitoid (Hymenoptera, Diptera, and Nematoda) species composition were indistinguishable between plantation and native forest. NMDS ordinations of (a)**,** host (final stress = 0.19), and (b)**,** parasitoid (final stress = 0.18) species composition and abundances at plantation and native training sites, with data pooled over all collections at each site. Numbers represent sites and letters represent forest type within site (N = native forest, P = plantation forest).

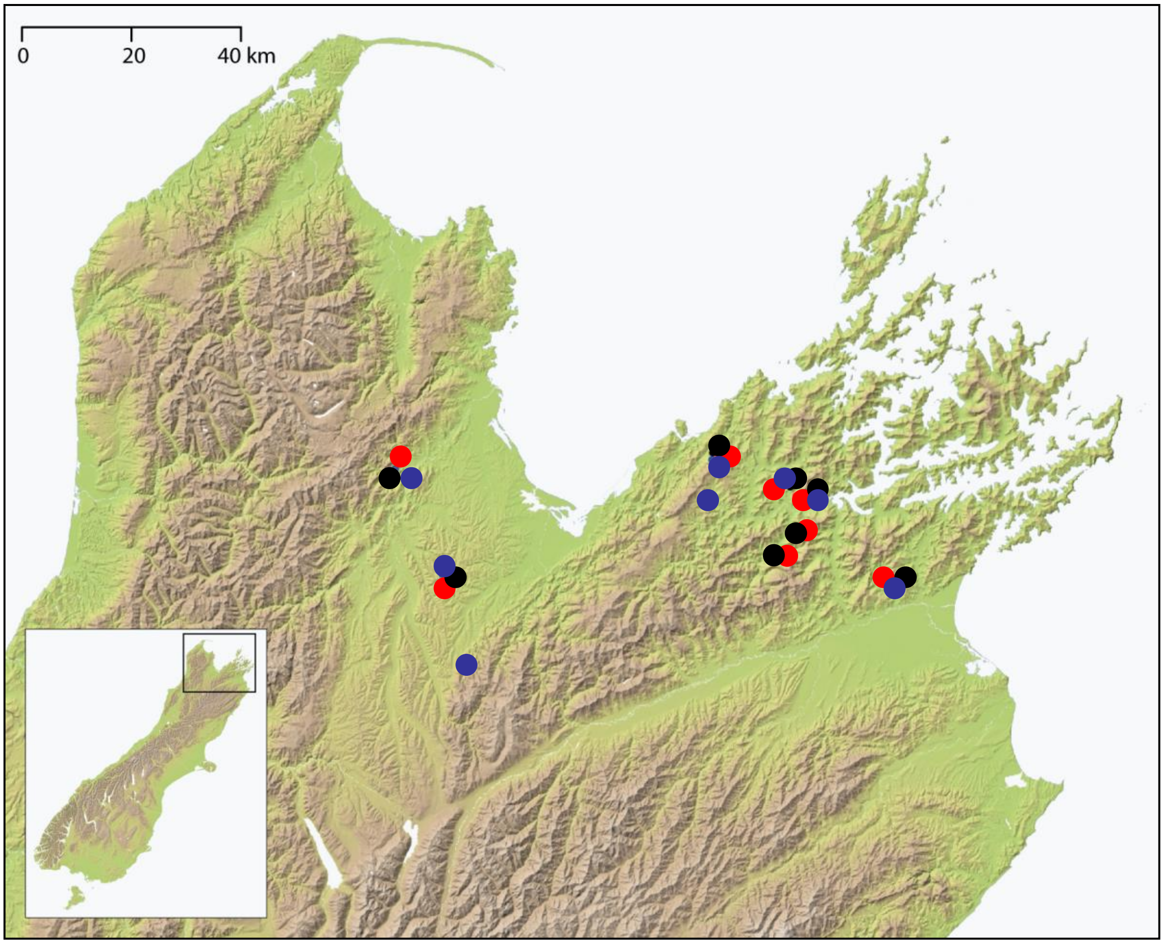

Supplementary Figure 4. Field site locations. All field sites were located in the Nelson/Marlborough region of South Island, New Zealand**.** Blue points represent training sites, and red (herbivore reduction treatment) and black (control) points represent validation sites. Map image credit: NASA's Earth Observatory.

Supplementary Tables

Supplementary Table 1. Herbivore reduction successfully reduced caterpillar abundance. Coefficients from the best-fitting model testing whether caterpillar abundance in plantation forest was affected by our herbivore reduction treatment.

	Estimate	Std. Error	z-value	P-value
Intercept (Control, Before)	3.4023	0.2370	14.35	**<0.0001**
Treatment (Herbivore reduction)	0.7457	0.3248	2.30	**0.0217**
Collection (After)	0.0829	0.3258	0.25	0.7992
Treatment (Herb. reduc.)*Collection (After)	-1.4659	0.4611	-3.18	**0.0015**

The model was a generalized linear mixed model with a Poisson error distribution (including the canonical log link function) and caterpillar abundance as the response variable. The predictors were collection (two levels: with the two before-herbivore-reduction collections pooled into one "before" sample vs. the two after-herbivore-reduction collections pooled into one "after" sample), treatment (herbivore reduction vs. control) and the collection x treatment interaction as fixed effects. Site, nested within block were included as random factors to account for the non-independence of the two treatments within each site, and the repeated measures at the before and after time steps at each site. Bold p-values indicate significance at $\alpha = 0.05$. The significant interaction between herbivore reduction treatment and collection ($p = 0.0015$) suggests that caterpillar abundance was initially greater at herbivore reduction sites (Treatment main effect) by chance (reduction vs. control treatments were allocated randomly), but after the herbivore reduction treatment, caterpillar abundance was significantly lower at treated sites than at control sites.

Supplementary Table 2. Herbivore reduction did not change parasitism rates in native forest. Coefficients from the best-fitting model testing whether parasitism rate in the native forest was affected by our herbivore reduction treatment in the adjacent plantation forest

	Estimate	Std. Error	z-value	P-value
Intercept (Control, Before)	-2.4415	0.2503	-9.755	**<0.0001**
Collection (After)	0.6886	0.1845	3.733	**0.0002**

The model was a generalized linear mixed model with a binomial error distribution (including the canonical logit link function) and parasitism rate in native forest as the response variable. The predictors were collection (two levels: with the two before-herbivore-reduction collections pooled into one “before” sample vs. the two after-herbivore-reduction collections pooled into one “after” sample), treatment (herbivore reduction vs. control) and the collection x treatment interaction as fixed effects. Site nested within block were included as random factors to account for the non-independence of the two treatments within each site, and the repeated measures at the before and after time steps at each site. Bold p-values indicate significance at $\alpha = 0.05$. The best model contained only collection; parasitism rates at both herbivore reduction and control sites were higher at the ‘after’ time step, though collection explained only 2.7% of the variation in parasitism rate in native forest ($R^2_{GLMM(m)} = 0.027$).

Supplementary Table 3. Expected parasitism rate predicted observed parasitism rate. Results of mixed-effects models testing whether expected parasitism rate, $E_{iA(t+1)}$, predicts observed parasitism rate, $O_{iA(t+1)}$

**Response variable:**	**Observed parasitism rate**			
	**Null model**	**Full model**		
**Fixed effects**	***b*** **[+/-SE]**	***b*** **[+/-SE]**	**z-value**	**P-value**
Intercept	**-1.266** [0.279]	**-1.826** [0.385]	-4.74	**<0.0001**
$E_{iA(t+1)}$	-	**2.288** [0.841]	2.72	**0.0065**
**VC for random effects**	**VC**	**VC**		
Site	0.322	0.377	-	-
**VC for fixed effects**	-	0.680	-	-
**PCV$_{[Site]}$**	-	-17.08%	-	-
**$R^2_{GLMM(m)}$**	-	15.64%	-	-
**$R^2_{GLMM(c)}$**	-	24.31%	-	-
**AIC**	83.1	77.3	-	-

The model was a generalized linear mixed effects model with a binomial error distribution (including the canonical logit link function), and block included as a random factor. Herbivore reduction treatment, forest, and all two-way and three-way interactions were removed from the full model during model selection, as were the forest within site nested random factors. AIC, Akaike Information Criterion; PCV, percentage change in variance; VC, variance components; $R^2_{GLMM(m)}$, marginal R^2; $R^2_{GLMM(c)}$, conditional R^2. Bolded coefficients and P-values are significant at $\alpha = 0.05$.

Supplementary Table 4. Predictions still worked when intraspecific effects were excluded. Results of mixed-effects models testing whether expected parasitism rate, $E_{iA(t+1)}$, could predict observed parasitism rate, $O_{iA(t+1)}$, with expected parasitism rates calculated excluding within-habitat intraspecific effects.

**Response variable:**	**Observed parasitism rate**			
	**Null model**	**Full model**		
**Fixed effects**	***b* [+/-SE]**	***b* [+/-SE]**	**z-value**	**P-value**
Intercept	**1.266** [0.279]	**-1.348** [0.318]	-4.24	**<0.0001**
$E_{iA(t+1)}$	-	**1.973** [0.833]	2.37	**0.0178**
$Habitat_A$		-0.781 [0.420]	-1.86	0.0627
**VC for random effects**	**VC**	**VC**		
Site	0.322	0.253		
**VC for fixed effects**	**VC**	**VC**		
$E_{iA(t+1)}$	-	0.592		
$Habitat_A$	-	0.042		
**$PCV_{[Site]}$**	-	21.43%		
**$R^2_{GLMM(m)}$**	-	15.19%		
**$R^2_{GLMM(c)}$**	-	21.25%		
**AIC**	83.1	77.5		

The model was a generalized linear mixed effects model, with a binomial error distribution (including the canonical logit link function), and block included as a random factor. Herbivore reduction treatment, the two-way interactions between $E_{ia(t+1)}$ and treatment, and $habitat_A$ and treatment, and the three-way interaction were removed from the full model during model selection, as were the forest within site nested random factors. AIC, Akaike Information Criterion; PCV, proportion change in variance; VC, variance components; $R^2_{GLMM(m)}$, marginal R^2; $R^2_{GLMM(c)}$, conditional R^2. Bolded coefficients and P-values are significant at $\alpha = 0.05$.

Supplementary Table 5. Initial parasitism rate did not predict observed parasitism rate. Results of mixed-effects models testing whether initial parasitism rate, I_{iAt}, predicts observed parasitism rate, $O_{iA(t+1)}$

**Response variable:**	**Observed parasitism rate**			
	**Null model**	**Full model**		
**Fixed effects**	***b* [+/-SE]**	***b* [+/-SE]**	**z-value**	**P-value**
Intercept	**-1.266** [0.279]	**-1.315** [0.465]	-2.83	**0.0047**
I_{iAt}	-	1.709 [1.537]	1.11	0.2662
Habitat_A		**-3.589** [1.627]	-2.21	**0.0274**
I_{iAt}*Habitat_A(Plantation)		12.633 [6.718]	1.88	0.0601
**VC for random effects**	**VC**	**VC**		
Site	0.322	0.381	-	-
**VC for fixed effects**	-		-	-
I_{iAt}	-	0.127	-	-
Habitat_A	-	3.262	-	-
I_{iAt}*Habitat_A(Plantation)	-	2.865	-	-
**$\text{PCV}_{[\text{Site}]}$**	-	-18.32%	-	-
**$R^2_{\text{GLMM(m)}}$**	-	35.01%	-	-
**$R^2_{\text{GLMM(c)}}$**	-	41.83%	-	-
**AIC**	83.1	75.5	-	-

The model was a generalized linear mixed effects model with a binomial error distribution (including the canonical logit link function), and block included as a random factor. Herbivore reduction treatment and all interactions involving herbivore reduction treatment were removed from the full model during model selection, as were the forest within site nested random factors. AIC, Akaike Information Criterion; PCV, percentage change in variance; VC, variance components; $R^2_{\text{GLMM(m)}}$, marginal R^2; $R^2_{\text{GLMM(c)}}$, conditional R^2. Bolded coefficients and P-values are significant at $\alpha = 0.05$.

Supplementary Table 6. Within-habitat data were sufficient for successful prediction. Results of mixed-effects models testing whether expected parasitism rate, (Log($E_{iA(t+1)}$ Within) and Log($E_{iA(t+1)}$ Cross) based on apparent competition between hosts within and across habitats predicts non-zero observed parasitism rate

**Response variable:**	**Observed parasitism rate**			
	**Null model**	**Full model**		
**Fixed effects**	***b*** **[+/-SE]**	***b*** **[+/-SE]**	**z-value**	**P-value**
Intercept	**-1.203** [0.282]	0.856 [0.764]	1.12	0.2628
Log($E_{iA(t+1)}$ Within)	-	**0.829** [0.292]	2.84	**0.0045**
**VC for random effects**	**VC**	**VC**		
Forest	0.558	0.343	-	-
**VC for fixed effects**	-	0.900	-	-
**PCV$_{[Forest]}$**	-	38.53%	-	-
**$R^2_{GLMM(m)}$**	-	19.86%	-	-
**$R^2_{GLMM(c)}$**	-	27.43%	-	-
**AIC**	74.9	68.2	-	-

The model was a generalized linear mixed effects model, with a binomial error distribution (including the canonical logit link function), and forest was included as a random factor. Cross-habitat expected parasitism rate was removed during model selection, as were the site within block random factors. AIC, Akaike Information Criterion; PCV, proportion change in variance; VC, variance components; $R^2_{GLMM(m)}$, marginal R^2; $R^2_{GLMM(c)}$, conditional R^2. Bolded coefficients and P-values are significant at $\alpha = 0.05$.

Supplementary Table 7. Cross-habitat data were also sufficient for successful prediction. Results of mixed-effects models testing whether expected parasitism rate based on apparent competition between hosts across habitats, Log($E_{iA(t+1)}$ Cross), predicts observed parasitism rate.

**Response variable:**	**Observed parasitism rate**			
	**Null model**	**Full model**		
**Fixed effects**	***b*** **[+/-SE]**	***b*** **[+/-SE]**	**z-value**	**P-value**
Intercept	**-1.203** [0.282]	0.370 [0.837]	0.44	0.6586
Log($E_{iA(t+1)}$ Cross)	-	**0.512** [0.250]	2.05	**0.0404**
**VC for random effects**	**VC**	**VC**		
Forest	0.558	0.864	-	-
**VC for fixed effects**	-	0.652	-	-
**PCV**$_{\textbf{[Forest]}}$	-	-54.84%	-	-
$\mathbf{R^2_{GLMM(m)}}$	-	13.56%	-	-
$\mathbf{R^2_{GLMM(c)}}$	-	31.55%	-	-
**AIC**	74.9	71.0	-	-

The model was a generalized linear mixed effects model, with a binomial error distribution (including the canonical logit link function), and forest was included as a random factor. AIC, Akaike Information Criterion; PCV, proportion change in variance; VC, variance components; $R^2_{GLMM(m)}$, marginal R^2; $R^2_{GLMM(c)}$, conditional R^2. Bolded coefficients and P-values are significant at $\alpha = 0.05$.

Supplementary Table 8. Predictions based on binary food web data worked less well. Coefficients from the best-fitting models testing whether a) expected parasitism rate, b) within- and cross-habitat expected parasitism rates, and c) cross-habitat expected parasitism rate, calculated using binary d_{iAjB}, could predict observed parasitism rate, $O_{iA(t+1)}$.

**Fixed effects**	**Estimate**	**Standard Error**	**z-value**	**P-value**
**a) Expected parasitism rate as a predictor**				
Intercept (Native, Control)	-2.4324	0.8394	-2.90	**0.0038**
$E_{iA(t+1)}$	1.9009	0.9931	1.91	0.0556
$Habitat_A$ (Plantation)	0.3934	0.5627	0.70	0.4844
Treatment (Herbivore reduction)	-0.0092	0.4972	-0.02	0.9853
$Habitat_A$ (Plantation)*Treatment (Herb. Reduc.)	-2.1577	0.9272	-2.33	**0.0200**
**b) Within- and cross-habitat expected parasitism rates as predictors**				
Intercept	0.0715	0.4612	0.16	0.8767
Log($E_{iA(t+1)}$ Within)	1.2986	0.3870	3.36	**0.0008**
**c) Cross-habitat expected parasitism rate as a predictor**				
Intercept	-0.5384	0.4105	-1.31	0.1896
Log($E_{iA(t+1)}$ Cross)	0.6810	0.2904	2.35	**0.0190**

Each model was a generalized linear mixed effects model with binomial error distribution (including the canonical logit link function), and block included as a random factor. P-values in bold are significant at $\alpha = 0.05$. **a)** The forest-within-site nested random factors were removed during model selection. Expected parasitism rate, herbivore reduction treatment, and $habitat_A$, as well as all interactions were included as fixed effects. Compare these binary results to the quantitative results in Table 3. **b)** Cross-habitat expected parasitism rate was removed during model selection, as were the forest-within-site random factors. Compare these binary results to the quantitative results in Supplementary Table 6. **c)** Compare these binary results to the quantitative results in Supplementary Table 7.

Supplementary Table 9. Expected parasitism rate predicted change in host abundance.

Coefficients from the best-fitting model testing whether expected parasitism rate, $E_{iA(t+1)}$, predicts change in focal host abundance, Δn_{iAt}

	Estimate	Std. Error	z-value	P-value
Intercept	22.300	7.658	2.912	**0.0086**
$E_{iA(t+1)}$	-42.808	14.245	-3.005	**0.0070**

The random factors (forest, site, and block) did not explain any of the variation in change in focal host abundance, so we used a linear model. The full model contained as predictors expected parasitism rate, forest, herbivore reduction treatment, and all interactions, but forest, herbivore reduction treatment, and all interactions were eliminated during model selection. Bold p-values indicate significance at α = 0.05. R^2 = 0.311.

Supplementary Table 10. Initial parasitism rate did not predict change in host abundance.

Coefficients from the best-fitting model testing whether initial parasitism rate, I_{iAt}, predicts change in focal host abundance, $\Delta n_{iAt.}$

	Estimate	Std. Error	t-value	P-value
Intercept	4.909	5.897	0.833	0.414

The random factors (forest, site, and block) did not explain any of the variation in change in focal host abundance, so we used a linear model. The full model contained as predictors initial parasitism rate, forest, herbivore reduction treatment and all interactions, but none of the fixed factors improved model fit over the intercept model.

Supplementary Table 11. Potential for cross-habitat indirect effects was not asymmetrical. Coefficients from a general linear model testing whether the potential for indirect effects, d_{iAjB}, varied in magnitude depending on the habitats of the host pair. 'N' and 'P' refer to native forest and plantation forest, respectively, such that, for example, 'PN' refers the situation where host *i* is in plantation forest and host *j* is in native forest. Bold p-values indicate significance at α = 0.05.

Coefficients	Estimate	Standard Error	t-value	P-value
Intercept (PN)	-3.645	0.106	-34.39	**<0.0001**
Habitat_{AB} NN	0.389	0.138	2.81	**0.0050**
Habitat_{AB} NP	0.277	0.150	1.85	0.0648
Habitat_{AB} PP	0.400	0.155	2.57	**0.0103**

Data for this analysis were from the regional metaweb (see Fig. 2), so sites were pooled, and habitat-specific species pairs were replicates (PN: n = 178; NN: n = 255; NP: n = 178; PP: n = 155).

Supplementary Table 12. Food web metrics for habitat-specific food webs. Food web metrics for regional quantitative food web data from training sites, but with food webs from native and plantation forests separated to allow comparison between forest types.

Forest type	Host Shannon Diversity	Parasitoid Shannon Diversity	Connectance	Interaction Diversity	Interaction Evenness
Native	2.46	2.99	0.075	4.05	0.90
Plantation	2.46	3.16	0.071	3.95	0.90

Quantitative food-web data were pooled over all collection dates and sites, but separated by forest type. Network structure was very similar between forest types. Shannon Diversity was calculated using the diversity function in the vegan package[2] in R. Network metrics were calculated using the networklevel function in the bipartite package[3] in R, using the 'sum' method for calculating interaction evenness.

Supplementary References

1. Frost, C. M., Didham, R. K., Rand, T. A., Peralta, G. & Tylianakis, J. M. Community-level net spillover of natural enemies from managed to natural forest. *Ecology* **96,** 193–202 (2014).
2. Oksanen, J. *et al.* vegan: Community Ecology Package. (2015). at <http://cran.r-project.org/package=vegan>
3. Dormann, C. F., Gruber, B. & Fründ, J. Introducing the bipartite Package: Analysing Ecological Networks. *R news* **8,** 8–11 (2008).
